# Taxonomic study of genus *Peucela* Ragonot, 1891 (Lepidoptera, Pyralidae) in China, with descriptions of three new species

**DOI:** 10.3897/zookeys.976.56402

**Published:** 2020-10-20

**Authors:** Mujie Qi, Xinghai Zuo, Houhun Li

**Affiliations:** 1 College of Life Sciences, Nankai University, Tianjin, China Nankai University Tianjin China

**Keywords:** key, morphology, Pyralinae, Pyraloidea

## Abstract

The genus *Peucela* Ragonot, 1891 from China is revised. Three species are described as new to science, *P. acutativalva***sp. nov.**, *P. baishanzuensis***sp. nov.**, and *P. nigra***sp. nov.** In addition, *P. olivalis***comb. nov.** is newly combined. Photographs of adults, and male and female genitalia are provided. A key to the species of *Peucela* in China is also provided.

## Introduction

The genus *Peucela* Ragonot, 1891 (Pyralidae, Pyralinae) was described with *Pyralis pallivittata* Moore, 1888 from India as the type species. Warren (1896) described *P. fumosalis* and *P. rubrifuscalis* from India. Later, the same author, Warren (1897) described *P. zonalis* from South Africa. Viette (1951) and Marion (1955) described *P. bourgini* and *P. ignealis* from Madagascar, respectively. Leraut (2010) established the new genus *Goateria* Leraut, 2010 and transferred *P. bourgini* to this genus. Leraut (2011) transferred *P. ignealis* to the genus of *Zitha* Walker, 1865. To date, the genus *Peucela* is comprised of three valid species, which occur in South Africa and Asia (Moore 1888; Warren 1896, 1897; Nuss et al. 2020). In this study, the genus *Peucela* from China is reviewed, including descriptions of three new species and one new combination. The generic characters of *Peucela* are given in detail, and a key to Chinese species based on male genitalia characters is provided.

## Material and methods

The examined specimens were collected with light traps and the dissections of genitalia were prepared by following the methods introduced by Li (2002). Wing venation preparations were carried out by following the protocol of Li and Zheng (1996). Specimens were examined using an Olympus SZX16 stereomicroscope. Images of adults and genitalia were captured with a Leica M205A stereomicroscope and a Leica DM750 microscope plus Leica Application suite 4.2 software. Terminology follows Slamka (2006) and Li and Li (2009). All specimens examined, including the types of the new species, are deposited in the Insect Collection of Nankai University (**NKU**), Tianjin, China.

## Taxonomy

### Genus Peucela

Taxon classificationAnimaliaLepidopteraPyralidae

Ragonot, 1891

A5D8ACB9-D700-5FD2-A39A-77F0BDDBDF34

Peucela Ragonot, 1891: 47. Type species: Pyralis pallivittata Moore, 1888.

#### Generic characters.

**Figures 1–3.**
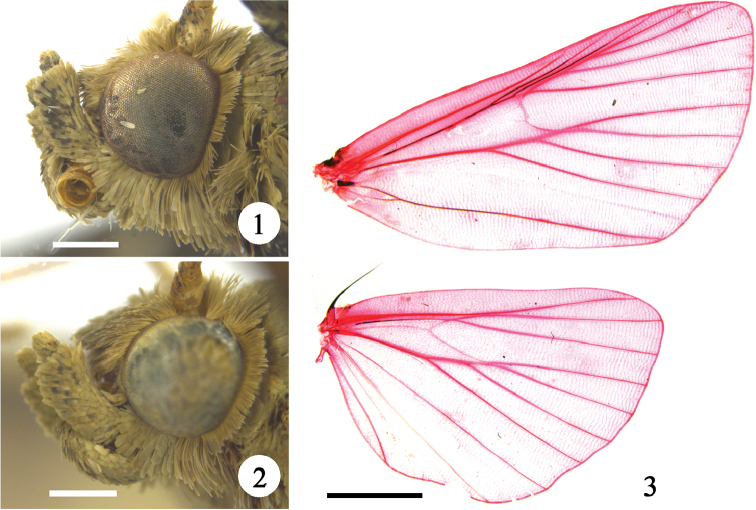
Morphology of *Peucela* spp. **1** head of *P. acutativalva*, sp. nov., ♂ **2** head of *P. acutativalva*, sp. nov., ♀ **3** venation of *P. nigra* sp. nov., slide No. QMJ15128w. Scale bars: 0.5 mm (**1, 2**); 2.5 mm (**3**).

**Adult.** Wingspan 19.0–26.5 mm. Frons rounded. Vertex covered with erect scales. Labial palpus upturned, third segment short and slightly porrect (Figs 1, 2). Maxillary palpus with three segments, usually as long as third segment of labial palpus and extending beyond its first segment. Antenna with scape dilated or ovate, flagellum filiform. Forewing ground color yellowish brown or grayish brown, with basal and terminal areas darker than median area. Hindwing concolorous with forewing, ante- and postmedial lines conspicuous. Venation (Fig. 3): forewing with 12 veins, R_3_ and R_4_ stalked from 2/5 length of R_3_, R_5_ stalked with R_3+4_ at basal 1/4, R_3+4+5_ and M_1_ connate at upper angle of cell, M_2_ and M_3_ connate at lower angle of cell, 1A+2A furcated at base; hindwing with 10 veins, Sc+R_1_ and Rs adjacent at middle of Sc+R_1_, Rs and M_1_ shortly stalked at upper angle of cell, M_2_ and M_3_ connate at lower angle of cell.

**Male genitalia.** Uncus trapezoidal or at least nearly trapezoidal basally. Gnathos with lateral arms bandlike, sinuous or straight; distal process short and hooked; two free basal extensions from base of gnathos, rod-shaped or band-shaped, sometimes enlarged or distally lobe-shaped. Valva broadest at base, narrowed towards apex; sacculus well developed. Juxta oval or shield-shaped. Saccus U-shaped, rounded at apex. Phallus medially curved, with basal part slightly enlarged, distal part cylindrical and granular on inner surface; cornutus thorn-shaped, sometimes basally or medially furcate.

**Female genitalia.** Papillae anales ovate. Apophyses posteriores shorter than apophyses anteriores. Antrum sclerotized; colliculum usually sclerotized, folded laterally. Ductus bursae slim, several times as long as corpus bursae, usually granular on inner surface anteriorly. Corpus bursae rounded, signum present or absent.

#### Diagnosis.

The genus *Peucela* superficially resembles *Fujimacia* Marumo, 1939 in having a similar wing pattern. It can be distinguished from *Fujimacia* by having the uncus without a basolateral process at the base (with one basolateral process on each side at base in *Fujimacia*), the gnathos having two rod-shaped or band-shaped basal extensions (without basal extension in *Fujimacia*), and the valva without a spine-like process at the apex (with a small spine-like process at the apex) in the male genitalia. *Peucela* is also similar to *Maradana* Moore, 1884, but it can be distinguished by having the uncus basally trapezoidal (conical in *Maradana*), the distal process of the gnathos rather tiny and hooked (distal process of gnathos usually about half as long as lateral arms in *Maradana*), and the phallus slightly enlarged basally (evenly cylindrical in *Maradana*) in the male genitalia.

### Key to the species of *Peucela* in China based on male genitalia

**Table:** 

1	Valva distally curved; basal extension of gnathos not enlarged or lobe-shaped distally	**2**
–	Valva distally not curved; basal extension of gnathos enlarged or lobe-shaped distally	**3**
2	Uncus trapezoidal; gnathoas with lateral arms about half as long as basal extensions; valva rounded at apex (Fig. 9)	***P. baishanzuensis* sp. nov.**
–	Uncus basally trapezoidal; gnathoas with lateral arms as long as basal extensions; valva triangular at apex (Fig. 8)	***P. acutativalva* sp. nov.**
3	Uncus with apex about 1/3 width of basal width; lateral arms of gnathos as long as its basal extension (Fig. 10)	***P. nigra* sp. nov.**
–	Uncus with apex about 1/2 width of basal width; lateral arms of gnathos about 1.5 times as long as its basal extension (Fig. 11)	***P. olivalis***

### Peucela acutativalva

Taxon classificationAnimaliaLepidopteraPyralidae

Qi & Li sp. nov.

E058A799-391A-5DB9-9C19-F43BD58E951A

http://zoobank.org/04E42A7F-C659-4545-B026-0A2C8C05F3B1

**Figures 4–7.**
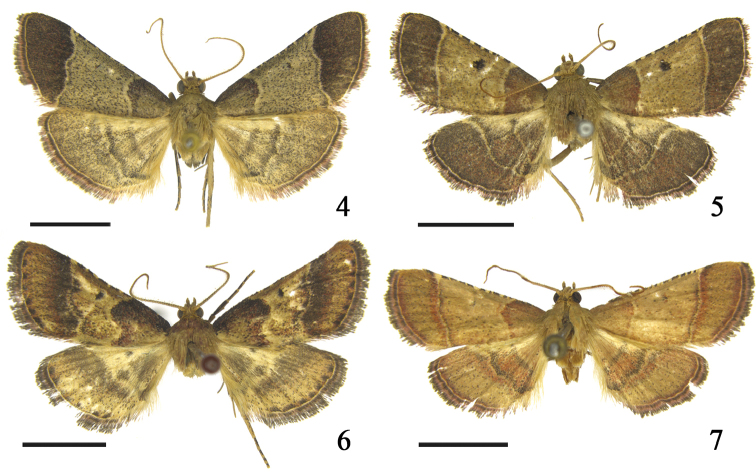
Adults of *Peucela* spp. **4***P. acutativalva*, sp. nov., holotype, ♂ **5***P. baishanzuensis*, sp. nov., holotype, ♂ **6***P. nigra* sp. nov., holotype, ♂ **7***P. olivalis*, ♂. Scale bars: 5.0 mm.

**Figures 8–11.**
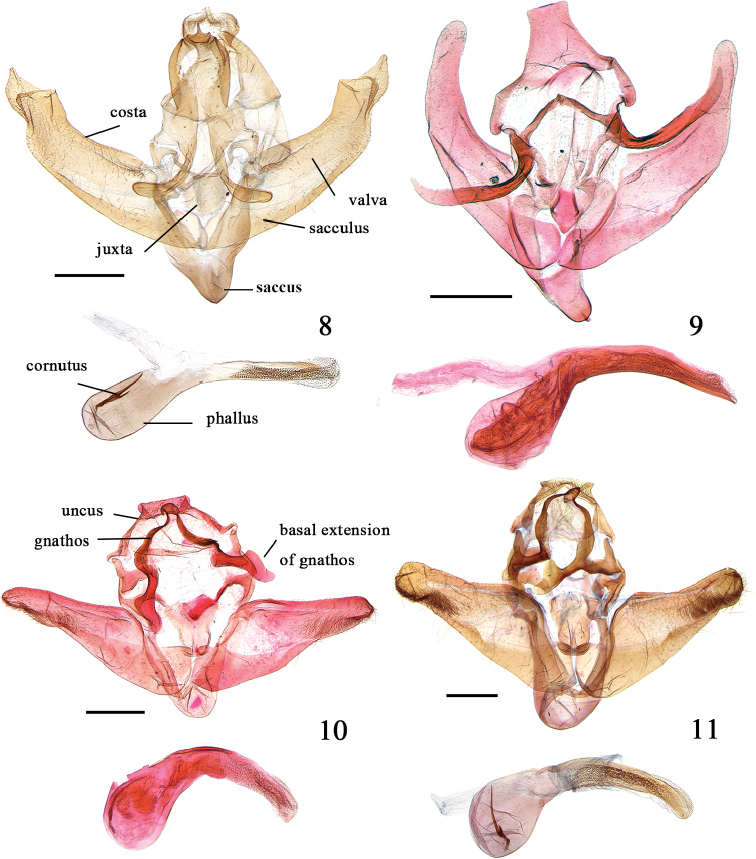
Male genitalia of *Peucela* spp. **8***P. acutativalva*, sp. nov., paratype, slide No. QMJ19023 **9***P. baishanzuensis*, sp. nov., holotype, slide No. LJ17121 **10***P. nigra* sp. nov., holotype, slide No. LJ17056 **11***P. olivalis*, slide No. QMJ19029. Scale bars: 0.5 mm.

**Figures 12–14.**
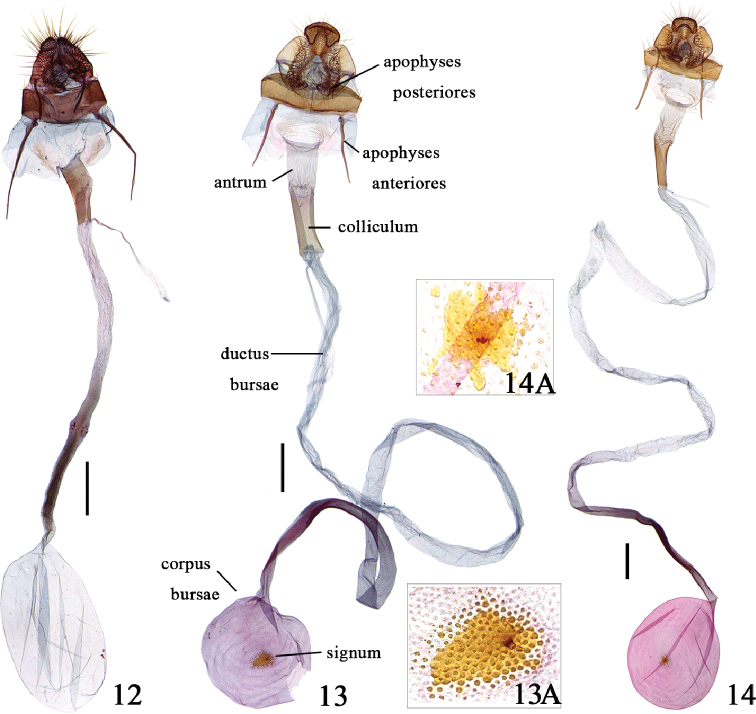
Female genitalia of *Peucela* spp. **12***P. acutativalva*, sp. nov., paratype, slide No. QMJ19048 **13***P. nigra* sp. nov., paratype, slide No. QMJ15128 **13A** Enlarged signum **14***P. olivalis*, slide No. QMJ15152 **14A** Enlarged signum. Scale bars: 0.5 mm.

Figs 1 , 2 , 4 , 8 , 12

#### Type material.

**China, Tibet: *Holotype***, ♂, Langjiu Village (28.40°N, 85.35°E), Gyirong County, 2772 m, 11.VII.2019, leg. Mujie Qi, Jiaqi Deng, genitalia slide No. QMJ19027.

***Paratypes***: 2 ♂♂, 2 ♀♀, same data as holotype, genitalia slides No. QMJ19024♂, QMJ19038♂, QMJ19045♀, QMJ19047♀; 2 ♂♂, 1 ♀, Zhangmu Town (27.98°N, 85.97°E), Nyalam County, 1961 m, 5–8.VII.2019, leg. Mujie Qi, Jiaqi Deng, genitalia slide No. QMJ19023♂, QMJ19050♂, QMJ19048♀; 1 ♀, Chongse Village (28.38°N, 85.36°E), Gyirong County, 2640 m, 14.VII.2019, leg. Mujie Qi, Jiaqi Deng, genitalia slide No. QMJ19039.

#### Diagnosis.

This species is similar to *P. pallivittata* (Moore, 1888) in the male genitalia, but it can be distinguished from the latter by the valva sharp at apex, the gnathos with the basal extension as long as the lateral arms, and the cornutus furcated at basal 1/3. In *P. pallivittata*, the valva is rounded at apex, the basal extension of the gnathos is about half the length of the lateral arms, and the cornutus is not furcated basally.

#### Description.

**Adult** (Fig. 4). Wingspan 22.0–23.5 mm. Frons and vertex yellowish brown. Labial palpus yellowish brown, slightly mixed with fuscous scales; first segment about 1/3 length of second; second segment upturned; third segment as long as first. Maxillary palpus with first segment as long as third; second segment with constriction medially, extending strongly beyond first segment of labial palpus; concolorous with labial palpus. Male antenna with scape ovate, flagellum ventrally ciliate. Patagium and tegula yellowish brown. Forewing grayish brown; basal area fuscous except for grayish brown at posterior half; terminal area fuscous, slightly mixed with reddish brown scales; costal margin fuscous except for median area interrupted with pale grayish brown spots; antemedial line pale grayish brown, from basal 1/4 of costa to basal 1/3 of dorsum, anterior half arched outwardly, posterior half concave inwardly; postmedial line concolorous with antemedial, edged with fuscous line on inner side, from distal 2/5 of costa to distal 1/8 of dorsum sinuously, with a distinct pointed convex between vein M_1_ and M_2_; median area sparsely suffused with fuscous scales, distal discoidal stigma fuscous; cilia fuscous at basal half, reddish brown at distal half. Hindwing concolorous with median area of forewing; ante- and postmedial lines pale grayish brown, both arched outwardly, outer side of antemedial and inner side of postmedial lines edged with fuscous lines; cilia fuscous at base, reddish brown distally. Foreleg fuscous except for tibia slight mixed with yellowish brown and each tip of tarsomere yellowish brown; midleg fuscous except for distal tibia and each apex of tarsomere yellowish brown; hindleg with femur fuscous, tibia and tarsus fuscous suffused with yellowish-brown scales, tarsus with scattered yellowish-brown scales, each tarsomere with apex yellowish brown.

**Male genitalia** (Fig. 8). Uncus with basal 3/5 trapezoidal, distal 2/5 oblong, with tiny triangular process at apex. Gnathos basally crescent-shaped, with a slim and upturned hook apically about 1/10 as long as uncus; basal extension rod-shaped, as long as lateral arms, slightly inflated distally. Valva broad basally, gradually narrowed towards triangular apex, ventral margin curved at apex, dorsal margin with flat triangular projection at distal 1/4 of valva. Sacculus broad at base, gradually tapered distally, about 3/5 as long as valva. Juxta shield-shaped. Saccus U-shaped, distal 1/4 slightly narrowed. Phallus with basal half oval, distal half cylindrical and granular; cornutus about 1/4 length of phallus, furcated at basal 1/3, furcated part about 1/5 as long as cornutus.

**Female genitalia** (Fig. 12). Papillae anales ovate. Apophyses anteriores gradually tapered toward apex, about 3 times as long as apophyses posteriores. Antrum sclerotized, as long as apophyses anteriores; colliculum well sclerotized, slightly folded laterally, about as long as or slightly shorter than antrum. Ductus bursae with basal half wrinkled, anterior half granular on inner side, about 3 times as long as corpus bursae. Corpus bursae rounded; signum absent.

#### Etymology.

The specific name is derived from the Latin *acutatus* (tapered) and *valva* (valva), in reference to the shape of the valva at the apex in the male genitalia.

#### Distribution.

China (Tibet).

### Peucela baishanzuensis

Taxon classificationAnimaliaLepidopteraPyralidae

Qi & Li sp. nov.

248BC8CF-11A5-5DE4-A606-466C7B5781BB

http://zoobank.org/EC73F1FE-785F-4EB0-8F0A-456C41A975B1

Figs 5 , 9

#### Type material.

**China, Zhejiang Province: *Holotype***, ♂, Baishanzu Nature Reserve (27°44'N, 119°10'E), Qingyuan County, 1149 m, 15.VIII.2016, leg. Qingyun Wang, Meiqing Yang, Ping Liu, genitalia slide No. LJ17121.

#### Diagnosis.

This species is superficially most similar to *P. olivalis*, but the male genitalia can be distinguished from that species in having the trapezoidal uncus not folded laterally at the apex, and the short and straight lateral arms of the gnathos about half as long as the basal extension, which is sharp and lacks an inflated triangular lobe distally. In *P. olivalis*, the uncus is basally semicircular, distally rectangular, and laterally folded at the apex; the lateral arms of the gnathos are arched and as long as the basal extension, which is inflated and distally lobe-shaped, and rounded at the apex.

#### Description.

**Adult male** (Fig. 5). Wingspan 25.0 mm. Frons and vertex yellowish brown. Labial palpus yellowish brown; first segment as long as third, about 1/4 length of second. Maxillary palpus yellowish brown, as long as third segment of labial palpus; extending slightly beyond first segment of labial palpus. Antenna of male with scape dilated; flagellum ventrally with cilia. Patagium and tegula pale grayish brown. Forewing grayish brown; basal area with anterior 1/3 fuscous along costa, posterior 2/3 pale reddish brown except basal area pale brown; median area pale brown, with scattered fuscous scales; terminal area fuscous, mixed with reddish brown scales; costal margin fuscous, interrupted by yellowish-brown spots at median area; antemedial line pale yellowish brown, arched outwardly from basal 1/4 of costa to basal 1/3 of dorsum; postmedial line concolorous with antemedial line, edged with fuscous line both on inner and outer sides, from distal 1/4 of costa to distal 1/6 of dorsum sinuously; distal discoidal stigma fuscous; cilia fuscous at base, distally reddish brown. Hindwing fuscous mixed with reddish brown scales; ante- and postmedial lines yellowish brown and sinuous; postmedial line with posterior half incurved inwardly, approximated to antemedial line at dorsum; cilia same as that in forewing. Foreleg fuscous except each apex of tarsomere yellowish brown; midleg with femur fuscous, tibia yellowish brown except basal half and apex fuscous, tarsus yellowish brown, suffused basally with fuscous scales; hindleg yellowish brown, femur suffused with fuscous scales, basal of spurs and tarsomere fuscous.

**Male genitalia** (Fig. 9). Uncus trapezoidal, flat at apex, laterally concave at distal 1/3. Gnathos with lateral arms slim and straight; distal process tiny and rhombic; basal extension upturned at basal 1/4, about twice as long as lateral arms of gnathos, knife-shaped at apex. Valva broad basally, gradually narrowed towards rounded apex; clasper flat, at below base of costa; sacculus gradually tapered distally, about 3/5 length of valva. Juxta with basal part sclerotized and rhombic, apical part membranous and rectangular. Saccus U-shaped, rounded at apex, about 1/3 length of sacculus. Phallus with basal part oval; distal part about 1.2 times as long as basal part, distally with granules; cornutus about 1/5 length of phallus.

**Female.** Unknown.

#### Etymology.

The specific name is derived from the type locality, Baishanzu Nature Reserve in Qingyuan County, Zhejiang Province.

#### Distribution.

China (Zhejiang).

### Peucela nigra

Taxon classificationAnimaliaLepidopteraPyralidae

Qi & Li sp. nov.

CF99B5F3-498B-559A-A76E-F64066F91B9F

http://zoobank.org/7C7F8047-04B4-4E42-BE04-7C0A915BBF5B

Figs 6 , 10 , 13

#### Type material.

**China, Yunnan Province: *Holotype***: ♂, Mt. Jizu (25.96°N, 100.39°E), Dali City, 2228 m, 27.VII.2014, leg. Kaijian Teng, Wei Guan, Xiuchun Wang, Shurong Liu, genitalia slide No. LJ17056.

***Paratypes***: 2 ♀♀, same data as holotype, genitalia slides No. QMJ15128, LJ17057; 1 ♀, Lingbaoshan Forestry Park, Nanjian County, 2338 m, 25.VIII.2015, leg. Kaili Liu, Jingxia Zhao, genitalia slide No. QMJ19026.

#### Diagnosis.

This species is similar to *P. olivalis* in the male and female genitalia, but it can be distinguished in the male genitalia by the uncus with the apical width about 1/3 of the basal width and the lateral arms of the gnathos as long as the basal extension and knife-shaped at apex; and in the female genitalia by the antrum as long as the colliculum and the corpus bursae about 1/10 as long as the ductus bursae. In *P. olivalis*, the apex of the uncus is about 1/2 width of its basal width, the lateral arms of the gnathos is 1.5 times as long as its basal extension, and is rounded at apex (Fig. 11); the antrum is longer than the colliculum and the corpus bursae is about 1/8 the length of ductus bursae (Fig. 14).

#### Description.

**Adult** (Fig. 6). Wingspan 23.0–26.5 mm. Frons and vertex covered with yellowish-brown scales. Labial palpus yellowish brown; second and third segments mixed ventrally with fuscous scales; first segment as long as third; second segment about 5 times as long as first; third segment porrect. Maxillary palpus yellowish brown, slightly shorter than third segment of labial palpus, extending slightly beyond first segment of labial palpus. Antenna with scape ovate, flagellum ventrally with cilia in male. Patagium reddish brown, with scattered fuscous scales; tegula grayish brown. Forewing yellowish brown; basal area with anterior half fuscous, mixed with reddish brown, posterior half grayish brown except for outer margin reddish brown and slightly mixed with fuscous; terminal area with basal half fuscous, outer margin reddish brown, apical half brown, slightly mixed with reddish brown and fuscous; terminal line fuscous with interrupted by reddish brown spots; costal margin fuscous except apex brown, interrupted by yellowish brown spots at median area; antemedial line pale yellowish brown, from basal 2/5 of costa to basal 1/3 of dorsum sinuously; postmedial line bounded by slim, fuscous line on inner side, waved from distal 2/5 of costa to distal 1/3 of dorsum; median area with scattered fuscous and reddish-brown scales, distal discoidal stigma fuscous; cilia fuscous except yellowish brown at base. Hindwing pale grayish brown, terminal area yellowish brown; ante- and postmedial lines yellowish brown, both sinuous; median area with anterior half yellowish brown, posterior half pale grayish brown, interrupted by yellowish-brown spots; cilia concolorous with forewing. Foreleg fuscous except each apex of tarsomere brown; midleg fuscous except behind spur and apex of tarsomere brown; hindleg fuscous, femur slightly mixed with yellowish scales, basal of tibia suffused with brown scales, each distal half of tarsomere brown.

**Male genitalia** (Fig. 10). Uncus with basal 4/5 semicircular; distal 1/5 rectangular, flat at apex, dorsally setose, ventrally with lobe-shaped process. Gnathos with lateral arms sinuous, distal process beaklike; basal extension as long as lateral arms, basal half straight, distal half dorsally with triangular dilatation. Valva basally broad, gradually narrowed towards truncated apex, setose at distal 2/5 of ventral margin of valva. Sacculus near subtriangular, about 2/3 as long as valva. Juxta with basal half ovate, distal part inverted trapezoidal. Saccus U-shaped, about 1/4 length of sacculus. Phallus with basal 1/3 oval; distal part about twice as long as base, apically gradually narrowed, with granules at distal 4/5; cornutus about 1/3 length of phallus.

**Female genitalia** (Fig. 13). Papillae anales with anterior 4/5 ovate, posterior 1/5 diamond-shaped. Apophyses anteriores gradually tapered toward apex, about 3 times as long as apophyses posteriores. Antrum about 4/5 length of apophyses anteriores; colliculum well sclerotized, laterally folded, about as long as apophyses anteriores. Ductus bursae about 10 times as long as corpus bursae, anterior 1/3 scobinate on inner surface. Corpus bursae rounded, scobinate on inner surface; signum consisting of numeral spinules, with short thorn at middle.

#### Etymology.

The specific name is derived from the Latin *niger* (black) in reference to the dark belt in the distal area of the forewing.

#### Distribution.

China (Yunnan).

### Peucela olivalis

Taxon classificationAnimaliaLepidopteraPyralidae

(Caradja, 1927) comb. nov.

D596F800-4B0A-5E77-BD5A-2007092089ED

Figs 7 , 11 , 14

Bostra olivalis Caradja, 1927: 44. TL: China.
Arippara indicator marginata (Walker, 1865): Leraut 2013: 60.

#### Material examined.

**Fujian**: 1 ♂, 1 ♀, Guadun, Mt. Wuyi, 1100 m, 28.VII.2008, leg. Weichun Li, Yongling Sun, Haiyan Bai, genitalia slide No. QMJ17021♂, QMJ17022♀; **Guangxi**: 1 ♂, Yangchang Forestry center, Leye County, 1160 m, 26.VII.2004, leg. Jiasheng Xu, genitalia slide No. QMJ16004; **Hainan**: 1 ♀, Mt. Diaoluo, 940 m, 31.V.2007, leg. Zhiwei Zhang, Weichun Li, genitalia slide No. LJ17086; 1 ♂, Jianfengling Nature Reserve (18.44°N, 108.52°E), Ledong County, 770 m, 29.V.2015, leg. Peixin Cong, Wei Guan, Sha Hu, genitalia slide No. QMJ19030; **Hunan**: 1 ♂, Zhangjiajie, 650 m, 7.VII.2001, leg. Houhun Li, Xinpu Wang, genitalia slide No. QMJ15197. **Sichuan**: 1 ♂, 2♀♀, Caoping Village (30.95°N, 103.32°E), Wenchuan County, 1557 m, 9–12.VII.2014, leg. Kaijian Teng, Wei Guan, Xiuchun Wang, Shurong Liu, genitalia slide No. QMJ16005♂, LJ17081♀, LJ16041♀; 3 ♀♀, Labahe Nature Reserve, Tianquan County, 1300 m, 28–29.VII.2004, leg. Yingdang Ren, genitalia slide No. LJ17078, QMJ15152, LJ17080; **Yunnan**: 1 ♂, Taiyanghe Nature Reserve, 1450m, 2.IX.2014, leg. Zhengguo Zhang, genitalia slide No. LJ16024; **Zhejiang**: 1 ♂, Chanyuansi, Mt. Tianmu, 350 m, 15.VIII.1999, leg. Houhun Li et al., genitalia slide No. WSS02136.

#### Diagnosis.

This species shares most of the diagnostic characters of the genus as follows: in male genitalia, gnathos with two free basal extensions at base and phallus medially curved, with basal part enlarged slightly; in female genitalia, colliculum cylindrical and sclerotized and ductus bursae several times as long as rounded corpus bursae and granular anteriorly. Adult (Fig. 7) with wingspan 19.5–22.0 mm. This species is superficially most similar to *P. baishanzuensis* sp. nov., and the differences between them are stated under *P. baishanzuensis* sp. nov. In addition, it resembles *P. nigra* sp. nov. in the male and female genitalia, and the differences between them are stated in the diagnosis of *P. nigra* sp. nov.

#### Distribution.

China (Fujian, Guangxi, Hainan, Hunan, Sichuan, Yunnan, Zhejiang).

## Supplementary Material

XML Treatment for Peucela

XML Treatment for Peucela acutativalva

XML Treatment for Peucela baishanzuensis

XML Treatment for Peucela nigra

XML Treatment for Peucela olivalis
